# The Genetic History of Indigenous Populations of the Peruvian and Bolivian Altiplano: The Legacy of the Uros

**DOI:** 10.1371/journal.pone.0073006

**Published:** 2013-09-11

**Authors:** José Raul Sandoval, Daniela R. Lacerda, Marilza S. A. Jota, Alberto Salazar-Granara, Pedro Paulo R. Vieira, Oscar Acosta, Cinthia Cuellar, Susana Revollo, Ricardo Fujita, Fabrício R. Santos, The Genographic Project Consortium

**Affiliations:** 1 Universidade Federal de Minas Gerais (UFMG), Belo Horizonte, MG, Brazil; 2 Universidad San Martin de Porres (USMP), Lima, Peru; 3 Universidad Mayor de San Andres (UMSA), La Paz, Bolivia; University of Florence, Italy

## Abstract

The Altiplano region of the South American Andes is marked by an inhospitable climate to which the autochthonous human populations adapted and then developed great ancient civilizations, such as the Tiwanaku culture and the Inca Empire. Since pre-Columbian times, different rulers established themselves around the Titicaca and Poopo Lakes. By the time of the arrival of Spaniards, Aymara and Quechua languages were predominant on the Altiplano under the rule of the Incas, although the occurrence of other spoken languages, such as Puquina and Uruquilla, suggests the existence of different ethnic groups in this region. In this study, we focused on the pre-Columbian history of the autochthonous Altiplano populations, particularly the Uros ethnic group, which claims to directly descend from the first settlers of the Andes, and some linguists suggest they might otherwise be related to Arawak speaking groups from the Amazon. Using phylogeographic, population structure and spatial genetic analyses of Y-chromosome and mtDNA data, we inferred the genetic relationships among Uros populations (Los Uros from Peru, Uru-Chipaya and Uru-Poopo from Bolivia), and compared their haplotype profiles with eight Aymara, nine Quechua and two Arawak (Machiguenga and Yanesha) speaking populations from Peru and Bolivia. Our results indicated that Uros populations stand out among the Altiplano populations, while appearing more closely related to the Aymara and Quechua from Lake Titicaca and surrounding regions than to the Amazon Arawaks. Moreover, the Uros populations from Peru and Bolivia are genetically differentiated from each other, indicating a high heterogeneity in this ethnic group. Finally, our results support the distinctive ancestry for the Uros populations of Peru and Bolivia, which are likely derived from ancient Andean lineages that were partially replaced during more recent farming expansion events and the establishment of complex civilizations in the Andes.

## Introduction

Since pre-Columbian times, the inhabitants of the Altiplano region in the South American Andes (between southern Peru, western Bolivia, and northern Chile and Argentina) have been engaged in agriculture, raising livestock and fishing. The Altiplano region or Collao Plateau lies in the central Andes, and presents an average height of about 3,750 meters above sea level, mean annual temperatures below 10°C, and total annual rainfall less than 1000 mm. Despite its harsh conditions, radiocarbon dating of artifacts suggested that the Altiplano was initially occupied by humans at approximately 3,700 years before the present [1].

At the time of arrival of the Spaniards in the 16^th^ century, most of this region was inhabited by Kollas, Lupakas, Pakaxes, and Carangas (mostly Aymara speaking clans or “señorios”) who were subjugated by the Inca Empire [2], [3], [4], [5]. However, other ethnic groups speaking unrelated languages such as Puquina and Uruquilla were also living around the Titicaca, Coipasa, and Poopo lake basins [6], [7], [8], [9], [10]. These languages gradually vanished during Spanish colonization, when Quechua and Aymara were imposed to facilitate administrative and evangelizing activities [3], [5]. Currently, most residents of the Altiplano speak Aymara (Jaqi-aru) and Quechua (Runa simi), “sister” languages of the Andean family that share about 20% of their vocabulary [5].

**Figure 1:**
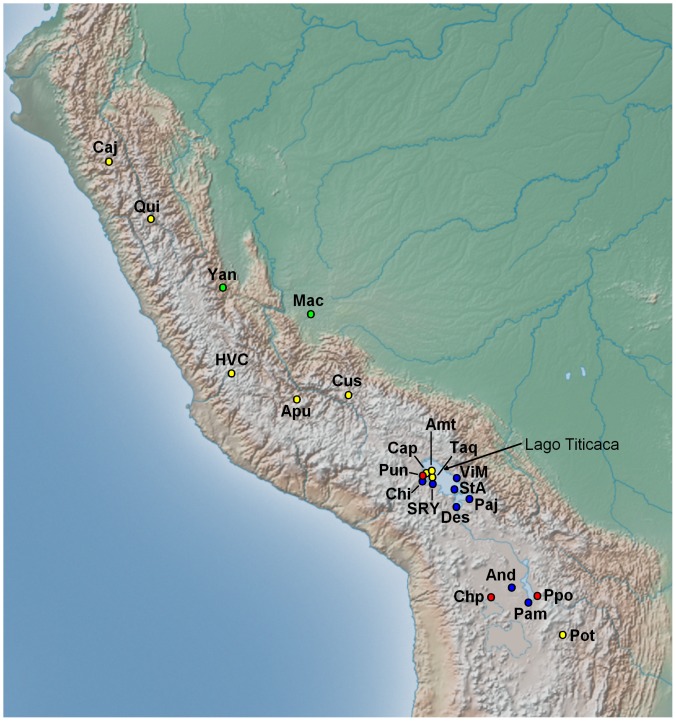
Map of the western South America showing the Andes and locations of the 22 Peruvian and Bolivian populations under study. Population codes are found in Materials and Methods (Sampling section). Yellow circles represent Quechua populations, blue circles represent Aymara, green circles represent Arawak, and red circles represent Uroś populations. A detailed map of the Andean Altiplano region can be seen in Figure S2.

Until the 16^th^ century, the Uruquilla speakers who were named Uros (or Urus, as they are called in Bolivia) were distributed along the aquatic axis comprising Lake Titicaca (Peru/Bolivia), the Azangaro and Desaguadero Rivers, Lake Poopo, the Lacajahuira and Lauca Rivers, and Lake Coipasa in Bolivia [2], [8]. The Uros population has diminished since this time, and is currently distributed in four different settlements dispersed along the aquatic areas of the Altiplano (Figure 1). In Bolivia, the Uru-Chipaya live near Lake Coipasa at the village of Santa Ana de Chipaya, the Uru-Poopo live in several villages close to Lake Poopo, and the Uru-Irohito (today with very few remnants) live on the banks of Desaguadero river, south of Lake Titicaca [7], [8]. In Peru, most of the Uros live in the Los Uros community, which is composed by the floating islands from Puno Bay of Lake Titicaca [8], [9].

The reduction of the Uros population has been suggested by linguists to have resulted from a gradual “transculturation” during pre-Inca, Inca and Spanish dominance, when many Uros were “puquinized”, “aymarized”, “quechuized”, and “castellanized” [6], [8], [9], [10]. Although the Uros’ original language (Uru or Uruquilla) was reportedly spoken in Puno Bay until 1929 [9], the current Peruvian population of approximately 219 families (∼2000 inhabitants) living in Lake Titicaca (Peruvian census at http://www.inei.gob.pe) speaks only Aymara and Spanish. Bolivian Uros (Uru-Chipaya, Uru-Poopo, and Uru-Irohito) are composed by about 2600 individuals (Bolivian census at http://www.ine.gob.bo), with the majority speaking only Aymara or Spanish [9]. However, about 1050 individuals from Santa Ana de Chipaya in Bolivia [12], [13] still speak their original language, Uru-Chipaya (likely derived from Uruquilla), although with a significant influence in the vocabulary from Aymara, Quechua, and more recently, Spanish language [8], [9].

According to some researchers, the Uros were the first settlers of the Andean Altiplano, yet their origin has been subjected to considerable academic debate [6], [8], [9], [13], [14]. Although in Aymara the term Uri means brave, wild, indomitable, and Uru means day, they are usually known by their neighbors as uma jaqe (men of the water) [8]. Actually, the Uros called themselves Qhas Qut suñi, which also means “men of the water” in the Uru (or Uruquilla) language [9]. However, some Native American groups that were not conquered by the Incas and Spaniards were also called Uros, like the Changos or “Uros de la costa” [6], [11]. Furthermore, the word Uro has been frequently used by the Incas and Conquistadores in distinct ways, referring to it as a language, ethnicity, a tax category or social status [8], [10].

Because of the complexity of their reported history and subjugation to other dominant societies since pre-Columbian times, the Uros have been the target of many research studies, particularly in the fields of anthropology and linguistics [7], [8], [9], [10], [13], [14], [15]. However, few genetic studies have been conducted with Uros communities. A previous study of HLA profiles [16] suggested an ancient connection between the Uros from Puno and Amazonian ethnic groups, following an old hypothesis of connection between Uruquilla (original language of the Uros) and the Arawak linguistic branch [7], [8]. Other genetic studies using mtDNA [17], [18] suggested that the Uros from Lake Titicaca still conserve part of their own ancient genetic background, while also forming a heterogeneous group. In any case, a larger survey of genetic data in many samples including other Uros’ subpopulations, Andean communities from the Altiplano, and Arawaks is still lacking. Such data would allow us investigating alternative hypotheses for the peopling of this region and to characterize genetic affinities of the current Uros’ communities inhabiting Peru and Bolivia.

Here, we generated and analyzed Y-chromosome and mtDNA diversity in 388 indigenous participants from Peru and Bolivia, focusing mainly on the pre-Columbian settlement of the Altiplano region and the ancestry of the Uros. Our results clarify the genetic relationships and population structure of the Peruvian and Bolivian Altiplano communities, as well as unveil the degree of kinship and shared ancestry between currently known Uros from Peru (Los Uros) and Bolivia (Uru-Chipaya and Uru-Poopo), and the major ethnic groups from the Andes, the Aymaras and Quechuas, and Arawaks from the Peruvian Amazon. With these data, we investigated three major questions: (i) Do the Uros from Bolivia and Peru share a recent common ancestry, or do they descend from distinct ancestors? (ii) How do the Uros relate to the neighboring Aymara and Quechua, and other populations from the Altiplano? and (iii) Do the Uros present any evidence of having close genetic kinship to Amazonian native communities of the Arawak family?

## Materials and Methods

### Ethics Statement

Ethical approval for the present study (The South American Genographic Project) was provided by the Brazilian National Ethics Commission (CONEP, Resolution number 763/2009), and by local ethical committees from Peru and Bolivia (USMP and UMSA, respectively). The project was explained to the volunteers after previous contact with indigenous confederations, authorities and/or community leaders, and in some cases in their indigenous languages. Signed informed consents for all subjects were obtained before collection of mouth swab samples. In most cases, collection expeditions occurred in the relatively isolated villages of the native participants, who were interviewed in order to assess the birthplace and ethnicity of their parents and grandparents, and to certify that at least three preceding generations of their ancestors had been living in the same locality. Relatives to the 3^rd^ degree were avoided, and men representing unique families were preferentially sampled to allow analyses with both Y-chromosome and mtDNA markers using a lower number of individuals, as women would only contribute to mtDNA analysis.

### Sampling

For the present study, we analyzed DNAs from 388 individuals residing in 22 sampling localities or communities (Figure 1, Table S1) that were divided into four major ethnic (or linguistic) groups. These included eight Aymara (n = 115; Chi = Chimu, SRY = Santa Rosa de Yanaque, ViM = Villa Molino, StA = Santa Ana, Paj = Pajchiri, Des = Desaguadero, And = Andamarca, Pam = Pampa Aullagas,), two Arawak (n = 29; Mac = Machiguenga, Yan = Yanesha), nine Quechua (n = 206; Caj = Cajamarca, Qui = Quinuabamba, HVC = Huancavelica, Apu = Apurimac, Cus = Cusco, Taq = Taquile, Amt = Amantani, Cap = Capachica, Pot = Potosi), and three Uros (n = 38; Pun = Los Uros-Puno, Chp = Uru-Chipaya, Ppo = Uru-Poopo) communities. An additional female sample from the Uru-Poopo community was included, and, therefore, the sample size was reduced to 387 for Y-chromosome analyses.

### Y-chromosome and mtDNA Analyses

DNA samples were extracted from buccal swabs using standard procedures. Samples were initially genotyped for the four more prevalent Y-SNPs autochthonous from South America, M130, M242, M346, and M3 [19], [20] using TaqMan assays (ABI) and a 7900HT Fast Real-Time PCR System (ABI). All samples belonging to the Native American Q haplogroup (lineages Q1a3* or Q-M346*, and Q1a3a or Q-M3, according to Karafet et al. [19]) and other haplogroups were further genotyped with 17 Y-chromosomal short tandem repeats (Y-STRs) using Y-filer™ Kit (ABI) and a 3130XL Genetic Analyzer (ABI) [20]. Fragment profiles of the Y-STRs were determined using the GeneMapper Software (v3.2, ABI). DNA controls supplied within the Y-filer Kit, as well as 20 Coriell DNA samples previously genotyped in University of Arizona, USA, were used to assess the quality and accuracy of the STR allele determinations.

Although the assignment of the two-repeat blocks of DYS385 could not be accurately made without further genotyping, it was suggested the shorter repeat allele was associated with DYS385a [21]. Anyway, we performed phylogenetic analyses with and without DYS385a and DYS385b data. In addition, DYS389b was calculated by subtracting DYS389I from DYS389II [22].

For all samples, the complete mtDNA control region (1122 bp, 16024-576 according to the revised Cambridge Reference Sequence (rCRS) [23]) was amplified with 15876-Forward and 639-Reverse primers, and sequenced using four oligonucleotides (15946-Forward, 132-Reverse, 16436-Forward, and 637-Reverse) [24], and the standard protocols for the 3130XL Genetic Analyzer (ABI) using Big Dye Terminator v. 3.1.

The sequence alignments were performed in relation to rCRS through SeqScape 2.6 (Applied Biosystems). Variable positions were determined and major haplogroup assignment was obtained by MitoTool [25] using as reference the rCRS and haplogroup prediction tool from Genographic Project (nnhgtool.nationalgeographic.com). Due to the phylogenetic uncertainty and alignment controversy, substitutions at nucleotide positions (np) 16182 and 16183, and indels at np 60, 72, 309, 315, 455, 519, 573, 16182, 16183 and 16193 were not used for phylogenetic and statistical analyses.

### Statistical Analyses

To reveal the genetic relationships among individuals we used the Median Joining algorithm through the program Network (www.fluxus-engineering.com), with and without weighting the Y-STRs or mtDNA haplotypes [26]. For Y-STR networks we used a weighting inversely proportional to twice the square root of the STR variance, yielding networks with low reticulation. For mtDNA networks, we used individual weights for variable sites based on the mutation number for each position, according to the Network manual (www.fluxus-engineering.com).

Analysis of Molecular Variance (AMOVA) was performed with Arlequin 3.5 [28], where *Fst* indices (*Rst* for STRs, and Φ*st* for mtDNA) were obtained to evaluate the genetic differentiation of the 22 communities. For estimation of a substitution model on mtDNA analyses, we used jModelTest 0.1.1 [27]. We used genetic distances linearized with population divergence times, converting *Rst* and Φ*st* distances into Reynolds’ coancestry coefficients in Arlequin, which were used in metric MDS analyses with GenAlEx [29], and non-metric MDS (nmMDS) calculated with PAST software (http://folk.uio.no/ohammer/past) to visualize population relationships in a bidimensional space. The goodness of fit stress values were estimated for nmMDS. The linearized Reynolds’ distances were also used in the Mantel test, comparing matrices of genetic and geographic distances (calculated with Geographic Distance Matrix Generator v1.2.3 - http://biodiversityinformatics.amnh.or/open_source/gdmg), and also in the spatial analysis using the Monmonier’s algorithm and Delaunay triangulation performed with the program Barrier [30]. In addition, the haplotype diversity indices and neutrality tests for distribution of mtDNA A2, B2, C1 and D1 haplotypes among the 22 populations were calculated with the Arlequin package under 10,000 permutations.

## Results

### Y-chromosome Results

After SNP genotyping samples from all 22 populations, we obtained Y chromosomal haplotypes from only two related Q lineages, Q-M346* (n = 24) and Q-M3 (n = 363). Because our main focus was investigating the origin of the Uros and all displayed only Q-M3 haplogroup, we selected 363 Q-M3 samples for individual level genetic analyses in phylogenetic networks, using 15 or 17 Y-STRs, with and without weighting. A complete list of Y-STR haplotypes is available in Table S2. Other population level analyses were performed with all native haplogroups.

**Figure 2:**
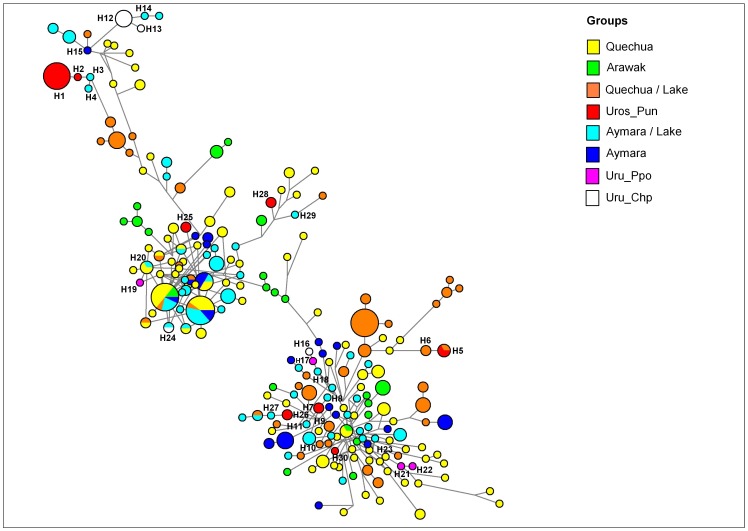
Median Joining network for Q-M3 STR haplotypes among 22 Peruvian and Bolivian populations. Different population groups are defined by distinct colors, where Aymara and Quechua communities from the border of the lakes (Titicaca and Poopo) are discriminated. The haplotypes (H#) composed of 15 Y-STRs are represented by circles with sizes proportional to numbers of individuals (H3 = 1, H1 = 13 males), and branch lengths are proportional to STR mutation steps (one repeat unit between H3 and H4). Haplotype names are according to the 15 Y-STRs (Table S2c).

Following the stepwise mutation model and parsimony criteria, we obtained a median-joining (MJ) phylogeographic network (Figure 2) of Y-chromosome STR haplotypes belonging to haplogroup Q-M3, by using 15 Y-STRs (excluding DYS385a and b, Table S2c) and weighting proportional to twice the inverse of square root variance. In Figure 2, we observed an isolated cluster of STR haplotypes very common among Peruvian Uros (red color) composed by H1 (n = 13) and H2 (n = 1). Other haplotypes appearing in this cluster were H3 and H4, which are closely related to the Uros haplotypes (H1, H2), and occurred in individuals from Santa Ana (StA), an Aymara community at the shores of Titicaca Lake, near the floating islands of Los Uros.

However, other Peruvian Uros’ haplotypes appeared in different clusters, all of which being more closely related to those found in communities around Lake Titicaca (Figure 1). For example, two individuals from Los Uros shared haplotype H5 with one Quechua from Amantani, which differed by one repeat unit from haplotype H6, which was also from Amantani. In the central agglomeration of the network, H7 from Los Uros (Pun) was very close to H8 from Santa Ana (Aymara), as well as to H9 from Amantani (Quechua), H10 from Pajchiri (Aymara) and H11 from Chimu (Aymara). Another Uroś haplotypes found in low frequency (H26, H25, H28, and H30) appeared scattered in the network.

Among Bolivian Uros, the Uru-Chipaya (Chp) haplotypes also formed a cluster bearing haplotypes H12 and H13, which was closely related to individuals bearing haplotype H15 from Pampa Aullagas (Pam) and H14 from Chimu (Chi), both neighboring Aymara communities. However, another haplotype H16 (Uru-Chipaya) did not belong to this cluster, but appeared genetically related to an Uru-Poopo (Ppo) haplotype H17. Both haplotypes (H16 and H17) were related to H18, which appeared in one Aymara from Pajchiri (Paj) on the border of Lake Titicaca. Moreover, haplotype H24 was shared between individuals from Uru-Chipaya and Santa Rosa de Yanaque (SRY), also at the shores of Lake Titicaca. In another cluster, H19 from one individual Uru-Poopo was closely related to H20 (one repeat unit difference) from Quechua of Potosi, very close to the Uro-Poopo locality. For two Uru-Poopo individuals, there was one difference between haplotypes H21 and H22, and those haplotypes were close to haplotype H23 (a Quechua from HVC). The major results of clustering and sharing of Peruvian and Bolivian Uros haplotypes can be also observed using all 17 Y-STRs and without weighting (Figure S1). Anyway, because of limited sample sizes of Bolivian Uros (Chipaya = 8, Poopo = 5 for mtDNA, and 4 for Y), some results should be taken with care.

**Figure 3:**
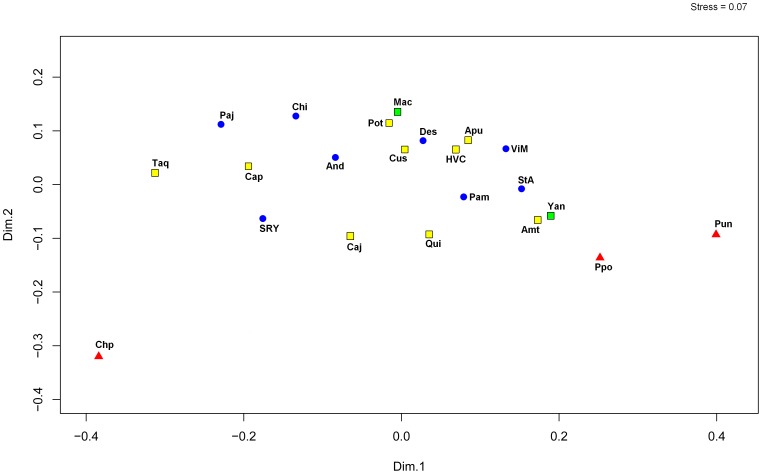
Non-metric MDS plot generated with PAST for Q-M346* and Q-M3 STR haplotypes among 22 Peruvian and Bolivian populations. It was used Reynolds’ *Rst* genetic distances among populations to build a bidimensional graphic with the Past software. Uros: red triangles, Aymara: blue circles, Quechua: yellow squares, and Arawaks: green squares.

In the nmMDS plot (Figure 3) of 17 Y-STR haplotypes for Q-M346* and Q-M3 lineages, we observed a compact cluster formed by all Aymara and Quechua and the two Arawak communities, while the three Uros communities appeared outside of this cluster. The Uru-Chipaya was the most isolated population, followed by Peruvian Uros and Uru-Poopo (Figure 3). We did not observe any clear geographic or ethnic/linguistic correlation, and the Arawaks appeared separated on different sides of the central cluster. Interestingly, the Quechua community of Taquile Island appears more isolated from the central cluster, which agrees with recently published data showing this community presents a relative isolation from other communities in Titicaca Lake [31]. Our nmMDS analyses show stress value = 0.07, below a 1% left-hand-tail cutoff value (stress = 0.293) for 22 objects in two dimensions considering tables from Sturrock and Rocha [32]. Although the configuration of the metric MDS calculated with GenAlex was different (figure not shown), the Bolivian and Peruvian Uros appear outside the major central cluster formed by Quechua and Aymara communities.

In the AMOVA results of different comparisons of Peruvian and Bolivian subpopulations (Table 1), we observed a complementary picture to the results observed at the individual level. When all major population groups were analyzed separately (Quechua, Aymara, Uros, and Arawaks), there was low differentiation among Aymara populations (*Rst* = 0.088), a moderate differentiation among Quechua groups (*Rst* = 0.136), and high differentiation among Arawaks (*Rst* = 0.245) and Uros (*Rst* = 0.478). The *Rst* comparisons in the three levels AMOVA also showed higher values when Uros were analyzed together Aymaras, Quechuas or Arawaks than analyses without them. However, there was little intergroup differentiation when subpopulations were clustered in four linguistic groups (4.41%). Besides a negative (*Fct* = −0.13) and non-significant differentiation (p = 0.9) was observed in the tree levels AMOVA between Uros and Arawaks (Table 1), which could be due to a very large differentiation among populations within Uros and Arawaks (*Fst* = 0.35, p<0.01). We also performed two levels AMOVAs to compare linguistic groupings with artificially merged subpopulations (Table S3a). Although these analyses indicated relatively small differentiation between Uros and Arawaks (*Rst* = 0.111), the latter appeared much closer to Aymara and Quechua (*Rst* = 0.066 and 0.045, respectively). The population pairwise *Rst* ´s between Peruvian and Bolivian Uros and other communities were all significant (p<0.05), except for Uro-Poopó that presented non-significant differentiation to Qui, Apu, Amt, ViM, Chi, Pam, and Pun likely due to its very small sample size (n = 4). Furthermore, the AMOVA between different population pairs (Table S3a) indicated that individual Uros populations were still more differentiated from Arawaks than the Aymara and Quechua, suggesting that within group heterogeneity was influencing these results.

**Table 1:** AMOVA results for 17 Y-STRs of the Q-M3 and Q-M346* lineages involving 22 Peruvian and Bolivian populations (n = 387).

Grouping	Among groups (%)	Among populations (%)	Within populations (%)	*Rst* indices***
1 group (all 22 communities)		18.81	81.19	0.188
4 groups (Quechua, Aymara, Uros, and Arawaks)	4.41**	15.65*	79.93	0.201
Uros		47.76	52.24	0.478
Aymara		8.83	91.17	0.088
Quechua		13.60	86.40	0.136
Arawaks		24.49	75.51	0.245
Uros/Aymara	14.39	16.32*	69.29	0.307
Uros/Quechua	15.02	16.23*	68.75	0.312
Uros/Arawaks	−13.07**	47.76*	65.31	0.347
Aymara/Quechua	−0.60**	12.02*	88.58	0.114
Aymara/Arawaks	3.39	10.50*	86.11	0.139
Quechua/Arawaks	0.10**	14.42*	85.48	0.145

* Between populations within groups.

** *Rct* (between groups), p-values>0.05.

*** p-values<0.01 (significant).

Non-significant correlation was observed between genetic distances (Reynolds’ *Rst*) and geographic distances (Km) in the Mantel test among the 22 communities (R^2^ = 0.013; p = 0.228), suggesting gene flow discontinuities. Moreover, the test considering geographic location and genetic distances through Barrier software showed that the Peruvian Uros, Taquile, Uru-Chipaya, Uru-Poopo, and Machiguenga communities were relatively isolated by gene flow barriers (Figure S2), in close agreement with the results of the MDS plot and AMOVAs.

### mtDNA Results

In a total of 388 individuals from autochthonous Peruvian and Bolivian populations, we identified 203 control region haplotypes belonging to four Native American haplogroups. No discrepancy in the haplogroup prediction was found between MitoTool and Genographic Project tool. Of these, 25 haplotypes corresponded to haplogroup A2 (n = 40), 133 haplotypes to haplogroup B2 (n = 285), 20 haplotypes to haplogroup C1 (n = 23), and 25 haplotypes to haplogroup D1 (n = 40). The haplotypes found in the Uros populations are listed in Table S4, and the complete set of variable sites of mtDNA sequences or haplotypes relative to the rCRS is shown in Table S5.

**Figure 4:**
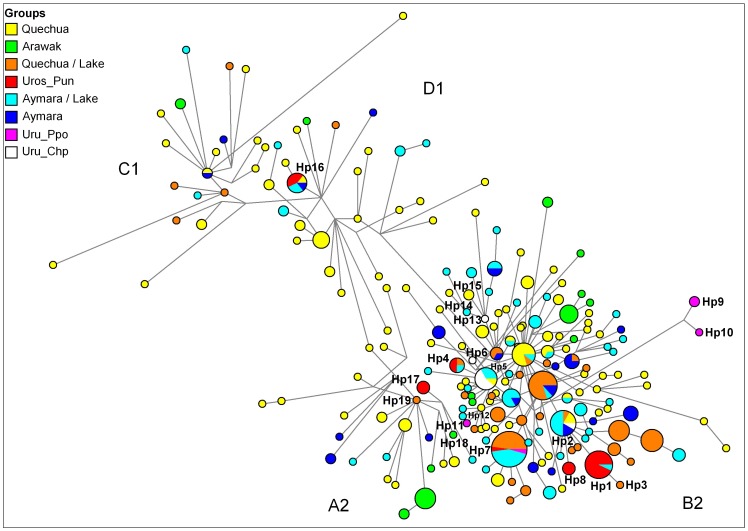
Median Joining network of mtDNA control region haplotypes found in 22 Peruvian and Bolivian populations. Different population groups are defined by distinct colors, where Aymaran and Quechuan communities from the border of the lakes (Titicaca and Poopo) are discriminated. The mtDNA haplotypes (Hp#) are represented by circles with sizes proportional to numbers of individuals (Hp10 = 1 individual), and branch lengths are proportional to nucleotide changes (1 mutation step between Hp1 and Hp3). Clusters of haplotypes into four mtDNA haplogroups (A2, B2, C1, D1) are indicated.

The distribution of mtDNA haplogroup frequencies in the 22 communities is shown in the Table S6. To characterize the genetic relationships among mtDNA haplotypes from Uros and other ethnic groups (at the individual level), we generated a MJ network from all control region haplotypes (Figure 4), and compared them through their site variants or SNPs (Table S4). In the MJ network, among B2 haplotypes, the most common haplogroup in the Altiplano (Figure 4), most of the Los Uros (Pun) individuals shared the haplotype Hp1 with one Aymara individual from Chimu (Chi), which was closely related to Hp3 from Taquile (Taq, Quechua), differing only at the np 146 (Tables S4 and S5). Also, the haplotype Hp1 was genetically related (two SNP changes at np 316 and 16170) to haplotype Hp2, and appeared in a cluster of individuals formed by Quechuas (Cus, Amt), Aymaras (Des, SRY, StA, And, Pam), and one Uru-Chipaya (Chp). The SNPs at np 316 and 16170 are recurrent, appearing also in single haplotypes of the C1 and A2 haplogroups, respectively (Table S5).

In other B2 clusters within the MJ network (Figure 4), we observed that haplotype Hp4 occurred in two Peruvian Uros, an individual from Capachica (Quechua) and another from Chimu (Aymara). Haplotype Hp4 was genetically close and probably derived from Hp5, which was shared among five Uru-Chipaya, three Aymara (Paj and Chi), and one Quechua from Potosi (Pot). Haplotype Hp6 from an Uru-Chipaya individual also appeared in this cluster, bearing a mutation at position 66 in relation to Hp5, which seemed to be the ancestral haplotype for Hp4 and Hp6. In another cluster, haplotype Hp7 was shared by 23 individuals from eight Altiplano communities (Amantani (n = 8), Yanaque (n = 5), Santa Ana (n = 4), Capachica (n = 2), Los Uros (n = 1), Taquile (n = 1), Villa Molino (n = 1), and Uru-Poopo (n = 1)) located along Lake Titicaca and Lake Poopo. This genetic pattern suggests a high maternal gene flow or recent common ancestry around this region. By contrast, Hp8 occurred in three individuals from Los Uros and was differentiated from all other Uros haplotypes, suggesting a relative degree of isolation of some maternal lineages among Peruvian Uros.

Among Bolivian Uros, three Uru-Poopo individuals appeared in an exclusive and highly differentiated B2 cluster (Figure 4) of related haplotypes Hp9 and Hp10. In other relationships, Hp11 from Uru-Poopo (Ppo) was close to Hp12 from Potosi (Pot), presenting a single base change at np 16172. Among the Uru-Chipaya, haplotype Hp13 (Chp) was very close to Hp14 from an Aymara individual from the border of Lake Titicaca (Des), but showed a single base difference at np 143 (Table S4). Interestingly, Hp13 was differentiated from Hp15 by two changes at np 16242 and 16324, which were carried by two individuals from Quinuabamba (Qui), a village located in the northern Andes (Ancash Department from Peru) and far away from Chipaya and Lake Titicaca.

Among sequences of haplogroup D1 (Figure 4), we observed that haplotype Hp16 was shared by Peruvian Uros (Pun), Aymaras from Chimu (Chi) and Andamarca (And), and a Quechua from Apurimac (Apu). Among haplogroup A2, we observed a cluster consisting of haplotypes Hp17 (Los Uros, n = 3), Hp18 (Machiguenga Arawaks, n = 1), and Hp19 (Quechua from Capachica, n = 1).

A maximum likelihood phylogenetic reconstruction (Figure S3) built with PhyML 3.0 (http://www.atgc-montpellier.fr/phyml) supports the close relationship of Uroś haplotypes discussed above, although the tree presents a branching topology that cannot be directly observed in the networks.

**Figure 5:**
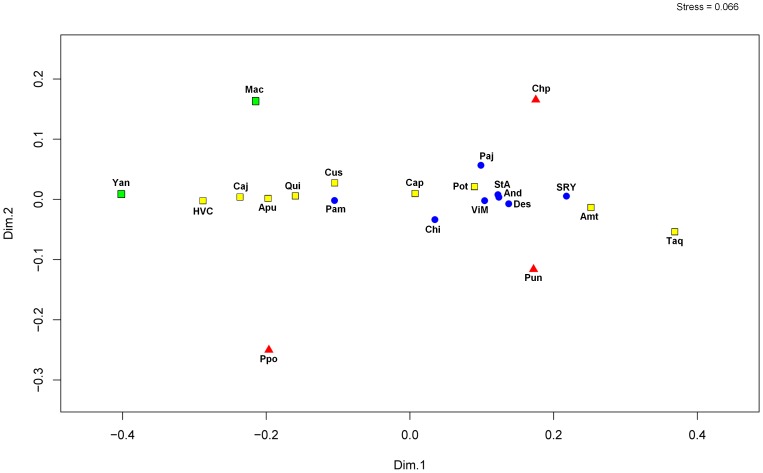
Non-metric MDS plot generated with PAST for mtDNA control region sequences for all haplogroups among 22 Bolivian and Peruvian populations. It was used Reynolds’ Φ*st* genetic distances among populations. Uros: red triangles, Aymaras: blue circles, Quechuas: yellow squares, and Arawaks: green squares.

The nmMDS plot generated from Reynolds’ linearized distances (Figure 5) showed that Arawaks (Machiguenga and Yanesha), Taquile and Bolivian and Peruvian Uros were separated from a compact cluster formed by other Quechua and Aymara populations. We also noted that Uru-Chipaya and Uru-Poopo appeared in opposite sides of the MDS plot. The Peruvian Uros appeared closer to communities of the shores of Titicaca Lake (Chi, SRY, ViM, Des, Amt), in the central cluster. In accordance with our previous Y-STR haplotype results (Figure 2), the Bolivian and Peruvian Uros, and Arawak populations appeared relatively differentiated among them (Figure 5), even though they belonged to the same major ethnic groups. Our nmMDS analyses show stress value = 0.066, below a 1% left-hand-tail cutoff value for 22 objects in two dimensions (stress = 0.293) considering tables from Sturrock and Rocha [32]. A similar configuration was obtained with metric MDS calculated with GenAlex (figure not shown), showing Bolivian and Peruvian Uros outside the major central cluster formed by Quechua and Aymara communities.

To characterize population structure and genetic diversity within and between the 22 Peruvian and Bolivian autochthonous communities under study, we carried out AMOVA tests following different population comparisons (Table 2). We performed the AMOVA runs using the TN93 substitution model with a gamma parameter (closest model available in Arlequin to TPM2uf +I+G obtained by the jModelTest algorithm) or using simple pairwise haplotype differences. Both generated similar results.

**Table 2:** AMOVA results for mtDNA control region sequences on the A2, B2, C1 and D1 haplogroups found among 22 Peruvian and Bolivian populations (n = 388).

Grouping	Among groups (%)	Among populations (%)	Within populations (%)	Φ *st* indices***
1 group (all 22 communities)		14.56	85.44	0.146
4 groups (Quechua, Aymara, Uros, Arawaks)	4.08	11.69*	84.23	0.158
Uros		30.98	69.02	0.310
Aymara		2.35	97.65	0.024
Quechua		13.69	86.31	0.137
Arawaks		25.22	74.78	0.252
Uros/Aymara	1.82**	7.21*	90.97	0.09
Uros/Quechua	1.80**	15.08*	83.12	0.169
Uros/Arawaks	10.89**	24.34*	64.77	0.352
Aymara/Quechua	−0.06**	10.01*	90.05	0.10
Aymara/Arawaks	18.57	5.26*	76.17	0.238
Quechua/Arawak	11.15**	13.05*	75.81	0.242

* Between populations within groups.

** *Fct* (between groups), p-value>0.05.

*** p-values<0.01 (significant).

When ethnic groups were analyzed separately by AMOVA (Table 2), the highest interpopulation differentiation was observed among Uros (Φ*st* = 0.310), followed by the Arawaks (Φ*st* = 0.252), Quechua (Φ*st* = 0.137), and Aymara *(*Φ*st* = 0.024). Thus, there was high heterogeneity among populations of Uros and Arawaks in comparison to Aymara and Quechua, a pattern similar to that produced by the AMOVA using Y-STR haplotypes. In the three level AMOVA results, we observed a lower interpopulation differentiation between Uros and Aymara (Φ*st* = 0.090) when compared to Uros and Quechua (Φ*st* = 0.169), but a particularly high difference between Uros and Arawaks (Φ*st* = 0.352). On the other hand, Aymara and Quechua populations were more closely related through their maternal lineages (Φ*st* = 0.090) than either was to Arawaks (Φ*st*>0.23). The population pairwise Φ*st’*s between Peruvian and Bolivian Uros, and other communities were all significant (p<0.05), except between Uru-Chipaya and Pajchiri (p = 0.06), We also conducted several two level AMOVAs between specific population pairs, or joining populations in linguistic groups (Table S3b). Compared to Y-STR analyses (Table S3a), mtDNA differentiation results were consistent with those of three level AMOVAs (Table 2), supporting a large differentiation between Uros and Arawak communities.

Haplotype diversity indices for B2 sequences in the 22 populations showed low values among Machiguenga (h = 0.417), Uros-Puno (h = 0.521), and Uru-Chipaya (h = 0.643), and this finding partially explains the relationships observed in the MDS plot (Figure 5). Tajima’s *D* and Fu’s *Fs* statistics showed significant negative values (p<0.05) among Potosi and Villa Molino populations, suggesting they have gone through demographic expansions in the past (Table S7).

We observed no correlation between Reynolds’ linearized distances of mtDNA data and geographic distances among the 22 populations using the Mantel test (R^2^ = 0.010; p = 0.248). However, the spatial analysis using genetic distances and geographic coordinates through Monmonier’s algorithm and Delaunay triangulation showed that the Machiguenga, Peruvian Uros, Uru-Poopo, Uru-Chipaya, and Taquile communities were isolated by geographic barriers (Figure S2) in close agreement with the MDS analyses, and previous results with Y-STR haplotypes.

## Discussion

This study focused on the genetic kinship and structure of Altiplano populations, and particularly that of the Uros, a self-identified ethnic affiliation used by different communities from Peru and Bolivia. Anthropologists and linguists [8], [9], [10], [13], [33] have always considered them to be an ethnic group separate from the Aymara and Quechua because of their different traditions, lifestyle and original language (Uru or Uruquilla).

Previous studies of Andean and Amazonian populations using dermatoglyphic patterns suggested that the Uru-Chipaya could be more related to Arawakan speakers of the Amazon forest than to Andean Quechua and Aymara [34]. An earlier study of mtDNA diversity [17] indicated that Uros from Puno were similar to Aymara from Anapia (an island from Lake Titicaca located in the frontier between Peru and Bolivia), although the presence of A2 among the Uros also suggested a differentiated genetic background, which in our results was related to an A2 haplotype from a Machiguenga Arawak (Figure 4). Our results suggest a remarkable heterogeneity within Uros populations, but also indicate that they possess a distinct genetic profile of maternal and paternal lineages in relation to other Andean populations.

Our analysis of Y-STR haplotypes from haplogroup Q-M3 suggests a distinctive ancestry of the Uros populations in the Altiplano of Peru and Bolivia. This distinctiveness may be related to their unique origin, peculiar demographic history and/or relatively lower admixture with their neighbors. At present, the Uru-Chipayas are living in an isolated location along the shores of Lake Coipasa, the Uru-Poopo live near Lake Poopo, and the Peruvian Uros live in the floating islands of the Lake Titicaca. As a result, all Uros communities are relatively distant from each other, as well. The Y-STR network (Figure 2) and population clusters observed in MDS (Figure 3) suggest higher gene flow between the Uros and surrounding populations at lake shores, but the Uros also possessed exclusive lineages that could be traced back to their unique ancestry.

In general, the MJ network of Y-STR haplotypes showed a remarkable divergence among current Peruvian and Bolivian Uros communities (Peruvian Uros, Uru-Chipaya, Uru-Poopo). It revealed some exclusive clusters of Uros, particularly among Peruvian Uros from Puno Bay. The few related haplotypes in this exclusive cluster (H1, H2, H3) are found in two Aymara individuals from Santa Ana, at the shore of Lake Titicaca, and this finding can be explained by unidirectional gene flow from Uros to Aymara or “Aymarization” of Uros, as suggested by linguists [6]. This trend may also be exemplified by other minor paternal haplotypes observed in Peruvian Uros, Uru-Chipaya and Uru-Poopo that were not seen in the isolated clusters, but were closely related to Aymara and Quechua lineages found in communities at shores of Lake Titicaca, such as Amantani, Santa Rosa de Yanaque, Santa Ana, Pajchiri, and Villa Molino. As shown in the MJ network (Figure 2), the Quechua and Aymara are not clearly differentiated, nor do they show any specific geographic clustering. Moreover, we observed remarkable differences between two Amazonian ethnic groups speaking different languages of the Arawak family, Machiguenga from Nuevo Mundo (Cusco department) and Yanesha from Oxapampa (Cerro de Pasco department), although their genetic profiles appear more closely related to those of Quechua and Aymara than to any Uros population.

The AMOVAs of both Y-chromosome and mtDNA haplotypes (Tables 1 and 2) indicated that Uros populations are differentiated from each other and also to other linguistic groups from the Altiplano, being more heterogeneous than the Aymara and Quechua, and particularly, the Arawaks. This pattern indicates that the Uros communities are similarly to autochthonous groups from the Amazon compared to less heterogeneous Andean populations, as suggested by a previous demographic model of evolution of South American native groups [35], [36]. These results further indicate that the ethnic and demographic histories of each particular population group are essential for explaining their current genetic profiles. Furthermore, drift and gene flow associated with relative isolation and geographic proximity, respectively, are important factors influencing the configuration of relationships between populations, as we have identified shared lineages and population similarities between Uros and neighboring populations of Quechua and Aymara.

The mtDNA results indicate that the Machiguenga (Mac) and Uru-Chipaya (Chp) are the most differentiated populations, followed by Peruvian Uros (Pun) and Uru-Poopo (Ppo). Among the Uros from Puno, the sharing of a mtDNA haplotype with an individual from Chimu community suggests that these individuals could be descendents of Aymarized “Uros”, as suggested by linguists [6], and other recent mtDNA analyses performed with the Chimu population [18]. The MJ network of B2 haplotypes shows that Andean Peruvian and Bolivian populations share several divergent lineages (Figure 4), while the Amazonian Machiguenga and Yanesha B2 lineages are not genetically similar (Figures 4 and 5). These results indicate that there has been historically high levels of gene flow and effective population size among Aymara and Quechua populations, a pattern previously observed with mtDNA control region and Y-STRs polymorphisms from Peru and Bolivia [18], [35], [36], [37].

Likewise, the cluster depicting the distribution of A2 haplotypes (Figure 4) suggests a common ancestral branch connecting Uros and Arawak mtDNAs, although they are also closely related to many haplotypes found in different Quechua communities from outside the region between Lake Titicaca and Lake Poopo. In addition, the wide distribution of D1 haplotypes going from Central Andes (Apu) towards south to the northern region of Lake Poopo (And) suggests either an ancient common ancestry before population splits or a large-scale and recent gene flow amongst groups in the region. In any case, because of the low prevalence of mtDNA lineages aside from B2, comparisons are limited due to small sample sizes.

In conclusion, the genetic evidence obtained through analyses of autochthonous paternal and maternal lineages from the Andean Altiplano showed that Uros from Puno Bay in Peru and Bolivian Uros communities of Uru-Chipaya and Uru-Poopo are heterogeneous groups, bearing genetic lineages derived from divergent ancestors when compared to most of the current Andean populations. Our results also indicate more gene flow with neighboring Andeans (Aymara and Quechua) than with more distant Andean communities or Arawakan speaking populations. Moreover, the results suggest the Uros could be derived from ancestral Andean stocks that were intermingled and partially replaced by lineages arriving with populations expanding due to a more recent farming expansion and posterior establishment of complex civilizations on the Andes.

## Supporting Information

Figure S1 **Median Joining network for 17-YSTR Q-M3 haplotypes among 22 Peruvian and Bolivian populations without weighting.** Different population groups are defined by distinct colors, where Aymara and Quechua communities from the border of the lakes (Titicaca and Poopo) are discriminated. The Y-STR haplotypes (H#) named according to Table S2a, are represented by circles with sizes proportional to numbers of individuals.
(TIF)

Figure S2 **Detailed map of the Altiplano region with four gene flow barriers detected among 22 Bolivian and Peruvian populations using the Barrier software.** The ranking order of barriers isolating populations is as follows: Peruvian Uros (Pun), Taquile (Taq), Uru-Chipaya (Chp), Uru-Poopo (Ppo), and Machiguenga (Mac, not shown) for Y-STR data analysis; and Machiguenga (not shown), Peruvian Uros, Uru-Poopo, Uru-Chipaya, and Taquile for mtDNA data analysis.
(TIF)

Figure S3 **A Maximum likelihood phylogenetic tree of all mtDNA control region haplotypes among Peruvian and Bolivian individuals (only topology is shown).** The haplogroups are indicated at the end of haplotype names. Red sequence names appear on the Uroś communities (Table S4) and are discussed on the text.
(TIF)

Table S1 **Description of the 22 Peruvian and Bolivian populations analyzed.**
(XLSX)

Table S2 **Y-STR haplotypes found among the 22 Peruvian and Bolivian populations.** a) individual 17 Y-STR haplotype data for Q-M3 and Q-M346* lineages; b) population data for Q-M3 Y-STR haplotypes; c) 15 Y-STR haplotypes.
(XLSX)

Table S3 **Two levels AMOVA results using 17 Y-STRs of Q-M3 and Q-M346 lineages (a) and four mtDNA haplogroups (b) involving comparisons of four linguistic groups without subpopulation division, and specific population pairs.**
(DOCX)

Table S4 **Control region mtDNA haplotypes and SNP variant positions found among Uros and related individuals from Peruvian and Bolivian populations.**
(DOCX)

Table S5 **Polymorphic sites in mtDNA CR sequences among Peruvian and Bolivian autochthonous individuals, determined by MitoTool program.**
(XLSX)

Table S6 **Distribution of mtDNA haplogroup frequencies (absolute values) among the 22 Peruvian and Bolivian populations.**
(DOCX)

Table S7 **Diversity indices and neutrality tests per population on 388 mtDNA CR sequences of A2, B2, C1 and D1 haplogroups.**
(DOCX)
