# Supplementary figures and images for: The Genetic History of Indigenous Populations of the Peruvian and Bolivian Altiplano: The Legacy of the Uros
Source: PLoS One. 2013 Sep 11;8(9):e73006. doi: 10.1371/journal.pone.0073006 (PMC3770642; doi:10.1371/journal.pone.0073006)

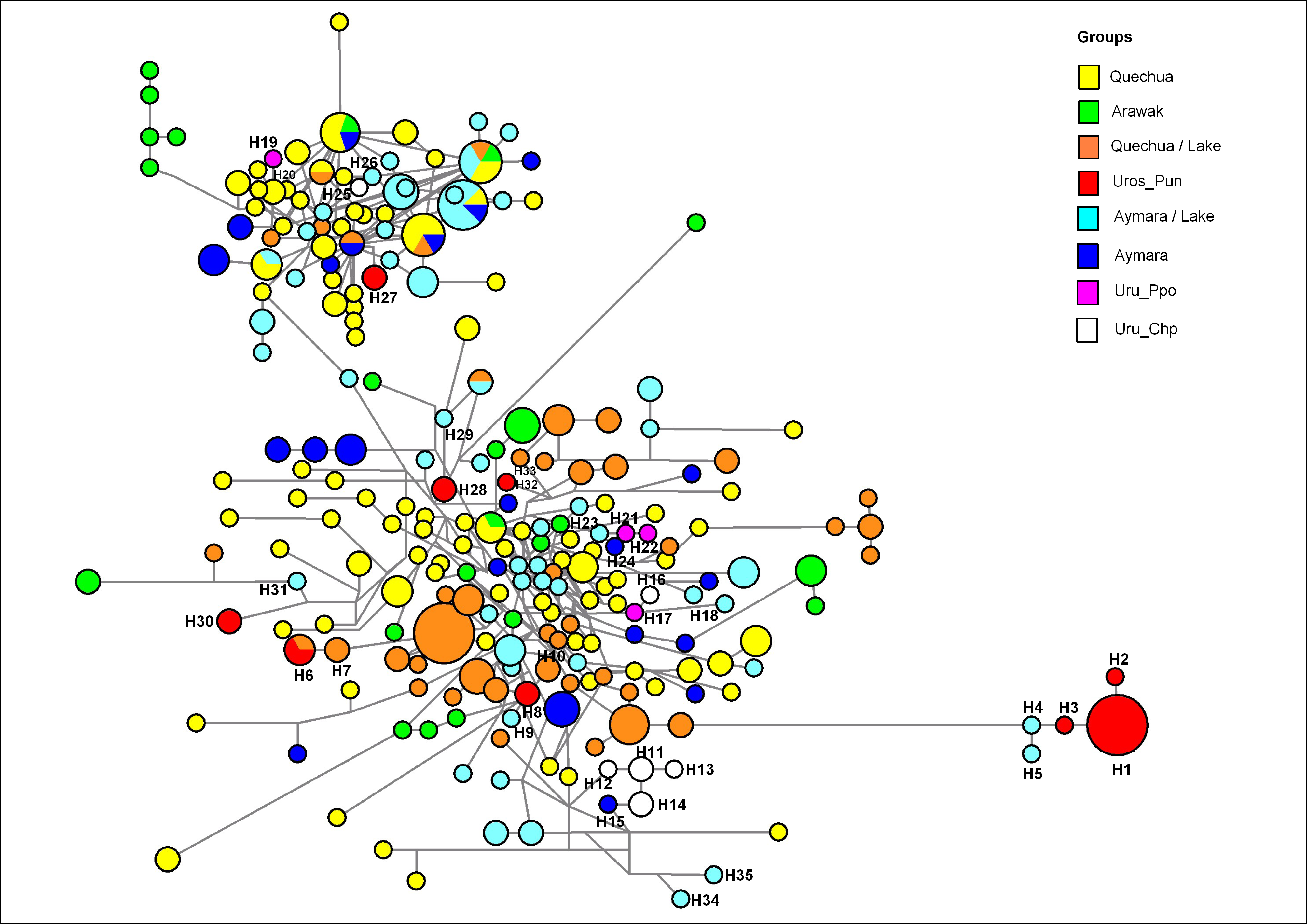

Supplement: Figure S1 — Median Joining network for 17-YSTR Q-M3 haplotypes among 22 Peruvian and Bolivian populations without weighting. Different population groups are defined by distinct colors, where Aymara and Quechua communities from the border of the lakes (Titicaca and Poopo) are discriminated. The Y-STR haplotypes (H#) named according to Table S2a, are represented by circles with sizes proportional to numbers of individuals. (TIF) [file pone.0073006.s001.tif]

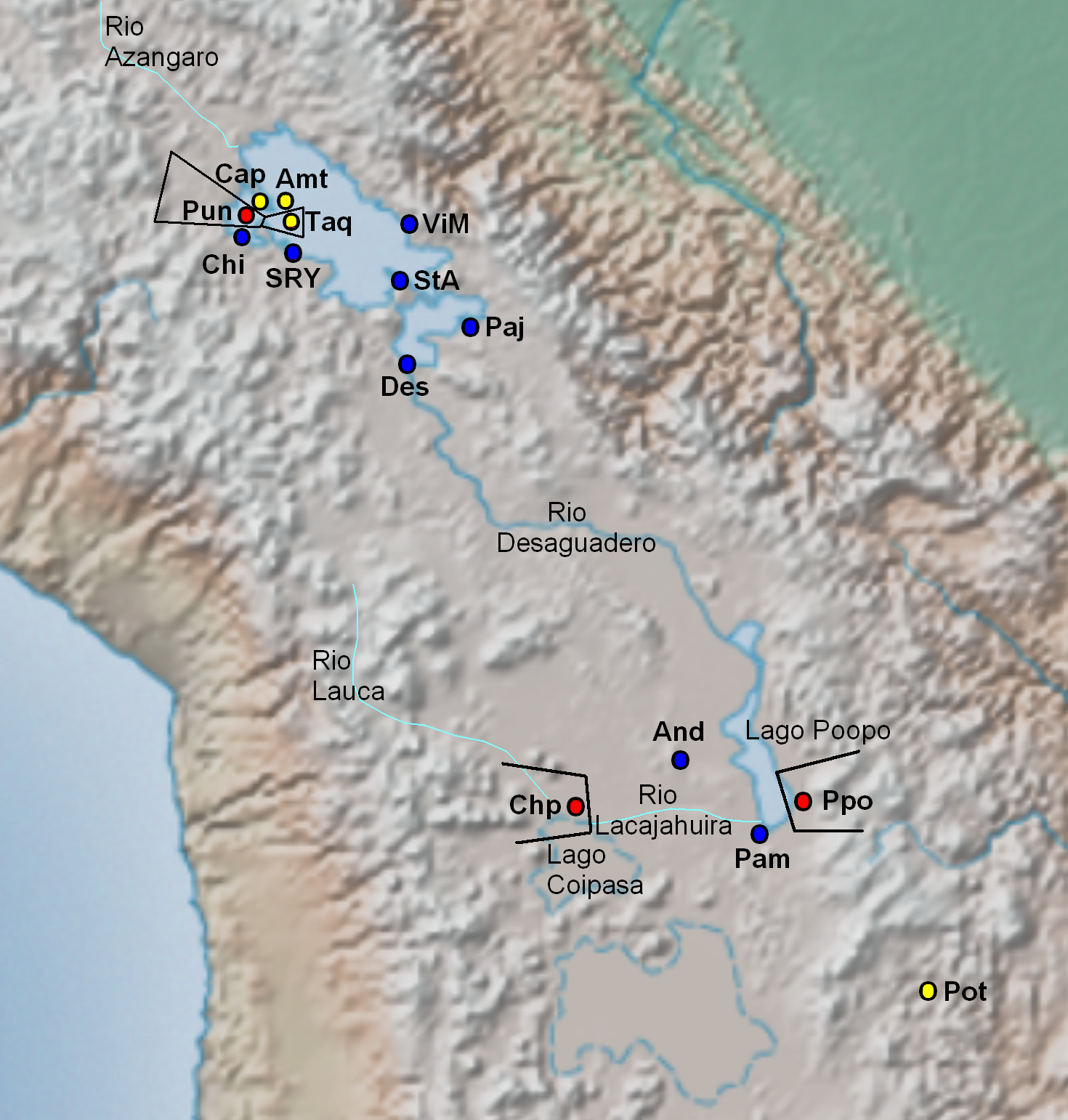

Supplement: Figure S2 — Detailed map of the Altiplano region with four gene flow barriers detected among 22 Bolivian and Peruvian populations using the Barrier software. The ranking order of barriers isolating populations is as follows: Peruvian Uros (Pun), Taquile (Taq), Uru-Chipaya (Chp), Uru-Poopo (Ppo), and Machiguenga (Mac, not shown) for Y-STR data analysis; and Machiguenga (not shown), Peruvian Uros, Uru-Poopo, Uru-Chipaya, and Taquile for mtDNA data analysis. (TIF) [file pone.0073006.s002.tif]

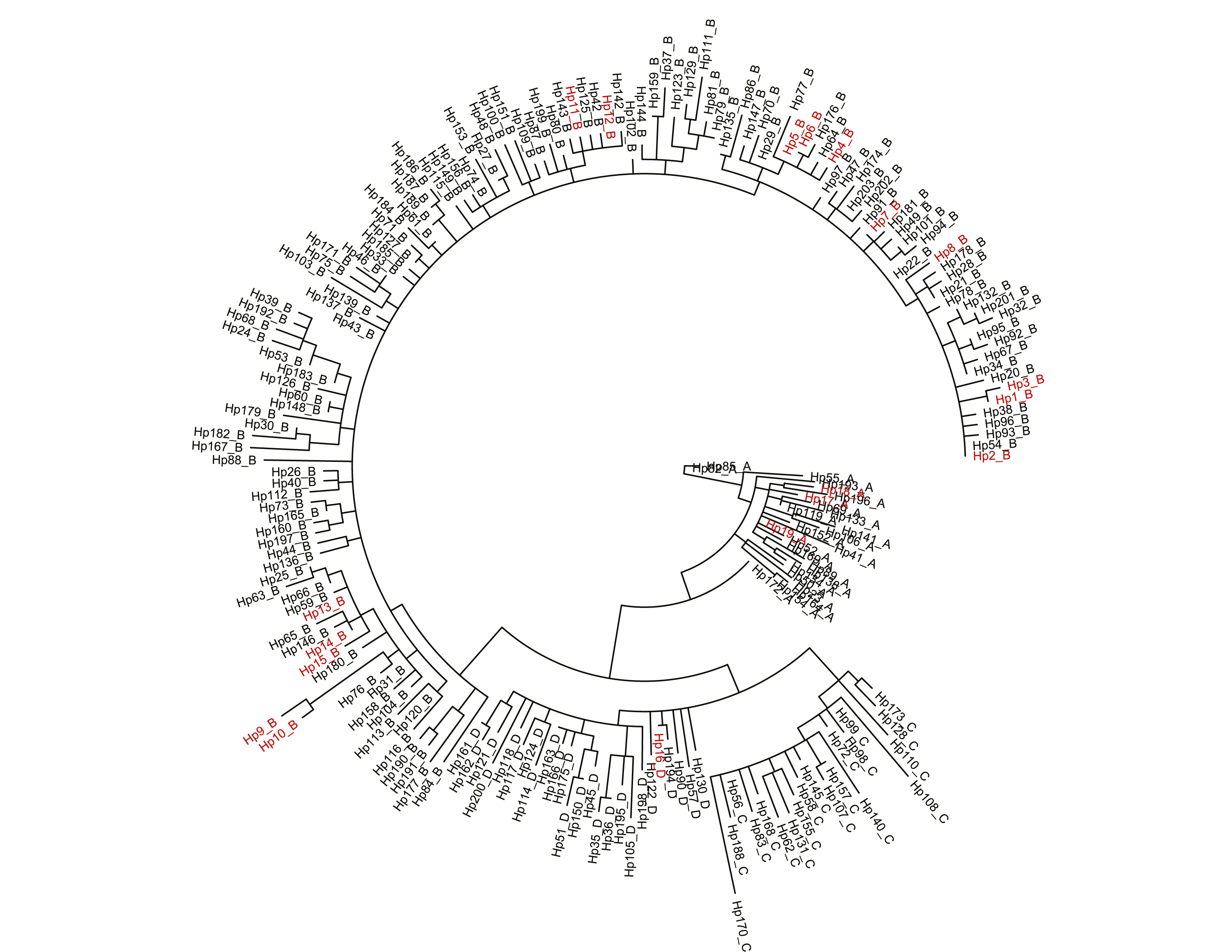

Supplement: Figure S3 — A Maximum likelihood phylogenetic tree of all mtDNA control region haplotypes among Peruvian and Bolivian individuals (only topology is shown). The haplogroups are indicated at the end of haplotype names. Red sequence names appear on the Uroś communities (Table S4) and are discussed on the text. (TIF) [file pone.0073006.s003.tif]
