# Supplementary material for: The Genetic History of Indigenous Populations of the Peruvian and Bolivian Altiplano: The Legacy of the Uros
Source: PLoS One. 2013 Sep 11;8(9):e73006. doi: 10.1371/journal.pone.0073006 (PMC3770642; doi:10.1371/journal.pone.0073006)
Supplement: Table S3 — Two levels AMOVA results using 17 Y-STRs of Q-M3 and Q-M346 lineages (a) and four mtDNA haplogroups (b) involving comparisons of four linguistic groups without subpopulation division, and specific population pairs. (DOCX) [file pone.0073006.s006.docx]

**Table S3a**. Two levels AMOVA results using 17 Y-STRs of Q-M3 and Q-M346 lineages involving comparisons of four linguistic groups without subpopulation division, and specific population pairs.

| Grouping | Among populations (%) | Within populations (%) | Rst | *p-values |
| --- | --- | --- | --- | --- |
| 1 group (Quechuas, Aymaras, Uros, and Arawaks) | 7.84 | 92.16 | 0.0784 | 0 |
| Uros / Aymaras | 19.2 | 80.8 | 0.192 | 0 |
| Uros / Quechuas | 19.6 | 83.4 | 0.196 | 0 |
| Uros / Arawaks | 11.1 | 88.9 | 0.111 | 0.001 |
| Aymaras/Quechuas | 0.9 | 99.1 | 0.009 | 0.022 |
| Aymaras /Arawaks | 6.6 | 93.4 | 0.066 | 0.001 |
| Quechuas /Arawaks | 4.5 | 95.5 | 0.045 | 0.002 |
| Uros Pun / Machiguengas | 36.2 | 63.8 | 0.362 | 0.0003 |
| Uros Pun /  Aymaras | 35.4 | 64.6 | 0.354 | 0 |
| Uros Pun /  Quechuas | 34.0 | 66.0 | 0.340 | 0 |
| Uros Pun /  Yanesha | 24.2 | 75.8 | 0.242 | 0.0005 |
| Urus BO** /  Machiguengas | 18.6 | 81.4 | 0.186 | 0.024 |
| Urus BO /  Aymaras | 8.3 | 91.7 | 0.083 | 0.011 |
| Urus BO /  Quechuas | 12.8 | 87.2 | 0.128 | 0.0007 |
| Urus BO /  Yanesha | 15.7 | 84.3 | 0.157 | 0.016 |
| Aymaras /  Machiguengas | 5.7 | 94.3 | 0.057 | 0.039 |
| Quechuas /  Machiguengas | 4.0 | 96 | 0.04 | 0.067 |
| Aymaras /  Yanesha | 13.2 | 86.8 | 0.132 | 0.0001 |
| Quechuas /  Yanesha | 11.0 | 89.0 | 0.110 | 0.0001 |
| Machiguengas/  Yanesha | 24.5 | 75.5 | 0.245 | 0.0002 |

* <0.05, significant. ** Bolivian Uros were merged for some comparisons.

**Table 3b**. Two levels AMOVA results using mtDNA control region sequences of the A2, B2, C1 and D1 lineages involving comparisons of four major ethnic groups, and specific population pairs.

| Grouping | Among populations (%) | Within populations (%) | φst | *p-values |
| --- | --- | --- | --- | --- |
| 1 group (Quechuas, Aymaras, Uros, and Arawaks) | 6.62 | 93.38 | 0.0662 | 0 |
| Uros / Aymaras | 3.97 | 96.03 | 0.0397 | 0.0016 |
| Uros / Quechuas | 6.20 | 93.80 | 0.0620 | 0 |
| Uros / Arawaks | 22.96 | 77.04 | 0.2296 | 0 |
| Aymaras/Quechuas | 1.21 | 98.79 | 0.0121 | 0.0071 |
| Aymaras /Arawaks | 20.11 | 79.89 | 0.2011 | 0 |
| Quechuas /Arawaks | 14.96 | 85.04 | 0.1496 | 0 |
| Uros Pun /  Machiguengas | 29.3 | 70.7 | 0.293 | 0.0001 |
| Uros Pun /  Aymaras | 8.6 | 91.4 | 0.086 | 0.0005 |
| Uros Pun /  Quechuas | 8.8 | 91.2 | 0.088 | 0.0002 |
| Uros Pun /  Yanesha | 33.0 | 67.0 | 0.330 | 0 |
| Urus BO** /  Machiguengas | 29.0 | 71.0 | 0.290 | 0 |
| Urus BO /  Aymaras | 6.3 | 93.7 | 0.063 | 0.009 |
| Urus BO /  Quechuas | 9.9 | 90.1 | 0.099 | 0.003 |
| Urus BO /  Yanesha | 35.0 | 65.0 | 0.350 | 0 |
| Aymaras /  Machiguengas | 16.8 | 83.2 | 0.168 | 0.0003 |
| Quechuas /  Machiguengas | 14.1 | 85.9 | 0.141 | 0.001 |
| Aymaras /  Yanesha | 31.7 | 68.3 | 0.317 | 0 |
| Quechuas /  Yanesha | 25.1 | 74.9 | 0.251 | 0 |

*< 0.05, significant. ** Bolivian Uros were merged for some comparisons.
