# Supplementary material for: The Genetic History of Indigenous Populations of the Peruvian and Bolivian Altiplano: The Legacy of the Uros
Source: PLoS One. 2013 Sep 11;8(9):e73006. doi: 10.1371/journal.pone.0073006 (PMC3770642; doi:10.1371/journal.pone.0073006)
Supplement: Table S4 — Control region mtDNA haplotypes and SNP variant positions found among Uros and related individuals from Peruvian and Bolivian populations. (DOCX) [file pone.0073006.s007.docx]

**Table S4**. Control region mtDNA haplotypes and SNP variant positions found among Uros and related individuals from Peruvian and Bolivian populations.

| Pop(n) | Hp | Variant / SNP | Hg |
| --- | --- | --- | --- |
| Pun (13);  Chi (1) | Hp1 | 73, 186, 263, **316**, 499, **16170**, 16188, 16189, 16217, 16519 | B2 |
| Des (3);  Cus (2);  SRY (2);  Amt (1);  StA (1);  And (1);  Chp (1);  Pam (1) | Hp2 | 73, 186, 263, 499, 16188, 16189, 16217, 16519 | B2 |
| Taq (1) | Hp3 | 73, 146, 186, 263, 316, 499, 16170, 16188, 16189, 16217, 16519 | B2 |
| Pun (2);  Cap (1);  Chi (1) | Hp4 | 63, 64, 73, 263, 499, 16188, 16189, 16217, **16464**, 16519 | B2 |
| Chp (5);  Paj (2);  Chi (1);  Pot (1) | Hp5 | 63, 64, 73, 263, 499, 16188, 16189, 16217, 16519 | B2 |
| Chp (1) | Hp6 | 63, 64, 66, 73, 263, 499, 16188, 16189, 16217, 16519 | B2 |
| Amt (8);  SRY (5);  StA (4);  Cap (2);  Pun (1);  Taq (1);  Ppo (1);  ViM (1) | Hp7 | 263, 499, 16188, 16189, 16217, 16519 | B2 |
| Pun (3) | Hp8 | 73, 152, 186, 263, 499, 16188, 16189, 16217, 16362, 16519 | B2 |
| Ppo (2) | Hp9 | 51, **56+C**, 73, 103, 139, 146, 151, 195, 263, 499, **16114**, 16189, 16217, 16294, 16359, 16519 | B2 |
| Ppo (1) | Hp10 | 51, 58, 73, 103, 139, 146, 151, 195, 263, 499, 16189, 16217, 16294, 16359, 16519 | B2 |
| Ppo (1) | Hp11 | 73, 204, 207, 263, 499, 16172, 16189, 16217, 16519 | B2 |
| Pot (1) | Hp12 | 73, 204, 207, 263, 499, 16189, 16217, 16519 | B2 |
| Chp (1) | Hp13 | 73, 146, 215, 263, 499, 16189, 16217, 16519 | B2 |
| Des (1) | Hp14 | 73, 143, 146, 215, 263, 499, 16189, 16217, 16519 | B2 |
| Qui (2) | Hp15 | 73, 146, 215, 263, 499, 16189, 16217, 16242, 16324, 16519 | B2 |
| Pun (3);  Chi (2);  And (1);  Apu (1) | Hp16 | 73, 263, 489, 16223, 16325, 16362, 16519, 16527 | D1 |
| Pun (3) | Hp17 | 64, 73, 146, 153, 235, 263, 16223, 16290, **16311**, 16319, 16362 | A2 |
| Mac (1) | Hp18 | 64, 73, 146, 153, 235, 263, **16189**, 16223, 16290, 16319, 16362, **16390** | A2 |
| Cap (1) | Hp19 | 64, 73, 146, 153, 235, 263, **16111**, 16223, 16290, 16319, 16362 | A2 |

Pop=population, (n)= sample size, Hp=Haplotype; Hg=Haplogroup. Variant/SNP positions are show using as reference the Cambridge Reference Sequence (see Mat. and Methods).
