# Supplementary material for: The Genetic History of Indigenous Populations of the Peruvian and Bolivian Altiplano: The Legacy of the Uros
Source: PLoS One. 2013 Sep 11;8(9):e73006. doi: 10.1371/journal.pone.0073006 (PMC3770642; doi:10.1371/journal.pone.0073006)
Supplement: Table S6 — Distribution of mtDNA haplogroup frequencies (absolute values) among the 22 Peruvian and Bolivian populations. (DOCX) [file pone.0073006.s009.docx]

**Table S6**. Distribution of mtDNA subhaplogroup frequencies (absolute values) among the 22 Peruvian and Bolivian populations.

| **Group/Pop** | **n** | **A2** | **B2** | **C1** | **D1** |
| --- | --- | --- | --- | --- | --- |
| **Quechua** |  | | | | |
| Caj | 19 | 1 | 9 | 2 | 7 |
| Qui | 10 | 1 | 6 | - | 3 |
| HVC | 26 | 4 | 8 | 4 | 10 |
| Cus | 36 | 8 | 22 | 4 | 2 |
| Apu | 10 | 1 | 5 | 2 | 2 |
| Pot | 29 | 2 | 25 | 1 | 1 |
| **Quechua/Lake** |  | | | | |
| Amt | 26 | - | 24 | 2 | - |
| Cap | 15 | 1 | 11 | 2 | 1 |
| Taq | 35 | - | 35 | - | - |
| **Aymara/Lake** |  | | | | |
| ViM | 7 | - | 7 | - | - |
| Chi | 16 | - | 11 | - | 5 |
| SRY | 18 | 1 | 16 | 1 | - |
| StA | 11 | - | 10 | 1 | - |
| Paj | 20 | 1 | 16 | - | 3 |
| Des | 11 | 1 | 10 | - | - |
| **Aymara** |  | | | | |
| And | 19 | 2 | 16 | - | 1 |
| Pam | 13 | 2 | 8 | 2 | 1 |
| **Uros** |  | | | | |
| Pun | 25 | 3 | 19 | - | 3 |
| Ppo | 5 | - | 5 | - | - |
| Chp | 8 | - | 8 | - | - |
| **Arawak** |  | | | | |
| Yan | 18 | 10 | 5 | 2 | 1 |
| Mac | 11 | 2 | 9 | - | - |
|  |  |  |  |  |  |
| Total | 388 | 40 | 285 | 23 | 40 |
