# Supplementary material for: The Genetic History of Indigenous Populations of the Peruvian and Bolivian Altiplano: The Legacy of the Uros
Source: PLoS One. 2013 Sep 11;8(9):e73006. doi: 10.1371/journal.pone.0073006 (PMC3770642; doi:10.1371/journal.pone.0073006)
Supplement: Table S7 — Diversity indices and neutrality tests per population on 388 mtDNA CR sequences of A2, B2, C1 and D1 haplogroups. (DOCX) [file pone.0073006.s010.docx]

**Table S7**. Diversity indices and neutrality tests per population on overall 388 mtDNA CR sequences of four haplogroups (A2, B2, C1, D1).

| Population | n | **Diversity index** | | | | **Neutrality’s test** | | | |
| --- | --- | --- | --- | --- | --- | --- | --- | --- | --- |
|  |  |  |  |  |  | Tajima | | Fu's Fs | |
|  |  | K | h | S | π | *D* | p | *Fs* | p |
| Cajamarca | 19 | 16 | 0.9766 | 53 | 0.009563 | -1.21138 | 0.10660 | -4.30150 | 0.04040 |
| Quinuabamba | 10 | 8 | 0.9556 | 36 | 0.009324 | -0.89235 | 0.19350 | -0.22656 | 0.41280 |
| Huancavelica | 26 | 19 | 0.9600 | 57 | 0.010234 | -0.96823 | 0.16520 | -3.71222 | 0.08550 |
| Apurimac | 10 | 10 | 1 | 44 | 0.011690 | -0.81849 | 0.21960 | -2.76964 | 0.05250 |
| Cusco | 36 | 30 | 0.9889 | 77 | 0.011037 | -1.26807 | 0.08790 | -12.80479 | 0.00110 |
| Taquile | 35 | 7 | 0.8000 | 9 | 0.002341 | 0.57685 | 0.75450 | 0.67156 | 0.66490 |
| Amantani | 26 | 14 | 0.8738 | 30 | 0.004349 | -1.39395 | 0.06530 | -3.37001 | 0.07450 |
| Capachica | 15 | 14 | 0.9905 | 46 | 0.008557 | -1.43849 | 0.06480 | -5.22521 | 0.01300 |
| Uros_Pun | 25 | 6 | 0.7067 | 24 | 0.006325 | 0.39555 | 0.70860 | 5.35032 | 0.96740 |
| Chimu | 16 | 10 | 0.9333 | 32 | 0.008303 | -0.17308 | 0.46770 | 0.11034 | 0.53120 |
| Yanaque | 18 | 11 | 0.9085 | 44 | 0.006706 | -1.59197 | 0.04190 | -0.81563 | 0.35640 |
| VillaMolino | 7 | 7 | 1 | 20 | 0.005301 | **-1.54260** | **0.03340** | **-2.62063** | **0.03920** |
| SantaAna | 11 | 7 | 0.8727 | 32 | 0.006698 | -1.48234 | 0.06370 | 0.63733 | 0.61100 |
| Pajchiri | 20 | 11 | 0.9158 | 40 | 0.007845 | -1.01485 | 0.15360 | 0.19728 | 0.55320 |
| Desaguadero | 11 | 9 | 0.9455 | 31 | 0.005946 | -1.73904 | 0.02630 | -1.94401 | 0.12940 |
| Yanesha | 18 | 9 | 0.8431 | 51 | 0.012632 | -0.12907 | 0.50180 | 3.22377 | 0.91390 |
| Machiguenga | 11 | 5 | 0.6182 | 23 | 0.005783 | -0.82427 | 0.21990 | 2.65416 | 0.89220 |
| Andamarca | 19 | 14 | 0.9649 | 39 | 0.006883 | -1.26145 | 0.09130 | -3.30406 | 0.07230 |
| PampaAullagas | 13 | 10 | 0.9487 | 49 | 0.011887 | -0.49239 | 0.33490 | -0.14328 | 0.45620 |
| Potosi | 29 | 23 | 0.9803 | 72 | 0.007812 | **-1.96387** | **0.00900** | **-9.64972** | **0.00200** |
| Uru_Poopo | 5 | 4 | 0.9000 | 16 | 0.007547 | 0.68730 | 0.73460 | 1.31990 | 0.67150 |
| Uru_Chipaya | 8 | 4 | 0.6429 | 7 | 0.001893 | -1.04453 | 0.18170 | 0.26095 | 0.51960 |

n=sample size

K=number of haplotypes

h=haplotypic diversity

S=number of polymorphic sites

π=nucleotide diversity (average over all loci)
